# Abundance of the Multiheme *c*-Type Cytochrome OmcB Increases in Outer Biofilm Layers of Electrode-Grown *Geobacter sulfurreducens*

**DOI:** 10.1371/journal.pone.0104336

**Published:** 2014-08-04

**Authors:** Camille S. Stephen, Edward V. LaBelle, Susan L. Brantley, Daniel R. Bond

**Affiliations:** 1 Department of Biochemistry and Molecular Biology, The Pennsylvania State University, University Park, Pennsylvania, United States of America; 2 BioTechnology Institute, University of Minnesota, St. Paul, Minnesota, United States of America; 3 Department of Geosciences, The Pennsylvania State University, University Park, Pennsylvania, United States of America; 4 Department of Microbiology, University of Minnesota, St. Paul, Minnesota, United States of America; Wageningen University, Netherlands

## Abstract

When *Geobacter sulfurreducens* utilizes an electrode as its electron acceptor, cells embed themselves in a conductive biofilm tens of microns thick. While environmental conditions such as pH or redox potential have been shown to change close to the electrode, less is known about the response of *G. sulfurreducens* to growth in this biofilm environment. To investigate whether respiratory protein abundance varies with distance from the electrode, antibodies against an outer membrane multiheme cytochrome (OmcB) and cytoplasmic acetate kinase (AckA) were used to determine protein localization in slices spanning ∼25 µm-thick *G. sulfurreducens* biofilms growing on polished electrodes poised at +0.24 V (vs. Standard Hydrogen Electrode). Slices were immunogold labeled post-fixing, imaged via transmission electron microscopy, and digitally reassembled to create continuous images allowing subcellular location and abundance per cell to be quantified across an entire biofilm. OmcB was predominantly localized on cell membranes, and 3.6-fold more OmcB was detected on cells 10–20 µm distant from the electrode surface compared to inner layers (0–10 µm). In contrast, acetate kinase remained constant throughout the biofilm, and was always associated with the cell interior. This method for detecting proteins in intact conductive biofilms supports a model where the utilization of redox proteins changes with depth.

## Introduction

The anaerobic respiratory strategy known as dissimilatory metal reduction likely evolved long before the Earth's atmosphere became aerobic [1], [2], and remains a significant process for geochemical cycling in sediments and subsurface environments [1], [3]. As reduction of metal oxides can support microbial oxidation of organic contaminants, and microbial reduction can alter the solubility of metals, dissimilatory metal reduction is also of involved in bioremediation and bioprecipitation of heavy metals [4]–[6]. A model metal-reducing bacterium capable of reducing both soluble and insoluble metals is *Geobacter sulfurreducens* [7]. Like most *Geobacter* strains, *G. sulfurreducens* can also use electrode surfaces as terminal electron acceptors, allowing generation of electricity [8]–[10].

When in contact with electrodes, *G. sulfurreducens* cells are capable of electron transfer from cell membranes to support growth. Daughter cells then grow as layers upon each other, connected by pathways conductive enough to transfer electrons tens of microns, allowing respiration by all cells in the biofilm [8], [11], [12]. Electron transfer by *G. sulfurreducens* electrode biofilms is dependent upon multiple extracellular proteins attached to cells [8], [9], [11], in contrast to representatives of the genus *Shewanella*, which rely on secreted FMN as a soluble electron shuttle for reduction of distant acceptors [8], [13]–[16].

Within *Geobacter* electrode biofilms, nutrient, pH, redox or electrical gradients may exist that affect cell physiology. For example, conduction of electrons through active biofilms appears to become limiting at distances 10–20 µm from the electrode surface, based on microelectrode [17], spectral [18], [19], source-drain experiments [12], [20], and confocal Raman spectroscopy [21]. A pH gradient can also exist across the biofilm, where the inner layers experience a lower pH [22]–[24].

The existence of these gradients has led to studies attempting to detect changes in gene expression across this narrow ∼20 µm window between the electrode surface and outer layers. Franks et al. [25] performed the first microarray analysis on *G. sulfurreducens* biofilm layers by microtoming sections into inner (0–20 µm) and outer (30–60 µm) leaflets. Of 146 genes differentially expressed [25] few differences were observed with genes linked to electron transfer, such as those encoding multiheme cytochromes, as well as subunits of Type IV pili. Immunogold labeling of the *G. sulfurreducens* extracellular cytochrome OmcZ suggested increased protein abundance close to the electrode (<5 µm) [26], but promoter fusion experiments visualizing *omcZ* expression were unable to detect any such gradient in *omcZ* expression, suggesting that differences in OmcZ could be due to mobility of this loosely attached cytochrome, or differences in cell density near the electrode [27].

For this work, a multiheme outer membrane cytochrome (OmcB) known to be regulated in response to environmental conditions [28]–[31] was selected as a target for an antibody-based approach for measuring changes in protein abundance within *Geobacter* anode biofilms. Acetate kinase was selected as a control for intracellular proteins. All measurements were performed using biofilms grown on polished anodes, to minimize variability in distance from the electrodes, and multiple high-resolution images were digitally reconstructed to obtain composite images spanning the entire biofilm for each labeling experiment. These data confirmed that direct labeling of resin-embedded *Geobacter* biofilms can be used to determine protein localization and detect changes in protein abundance throughout a biofilm.

## Results

### Biofilm growth

**Figure 1:**
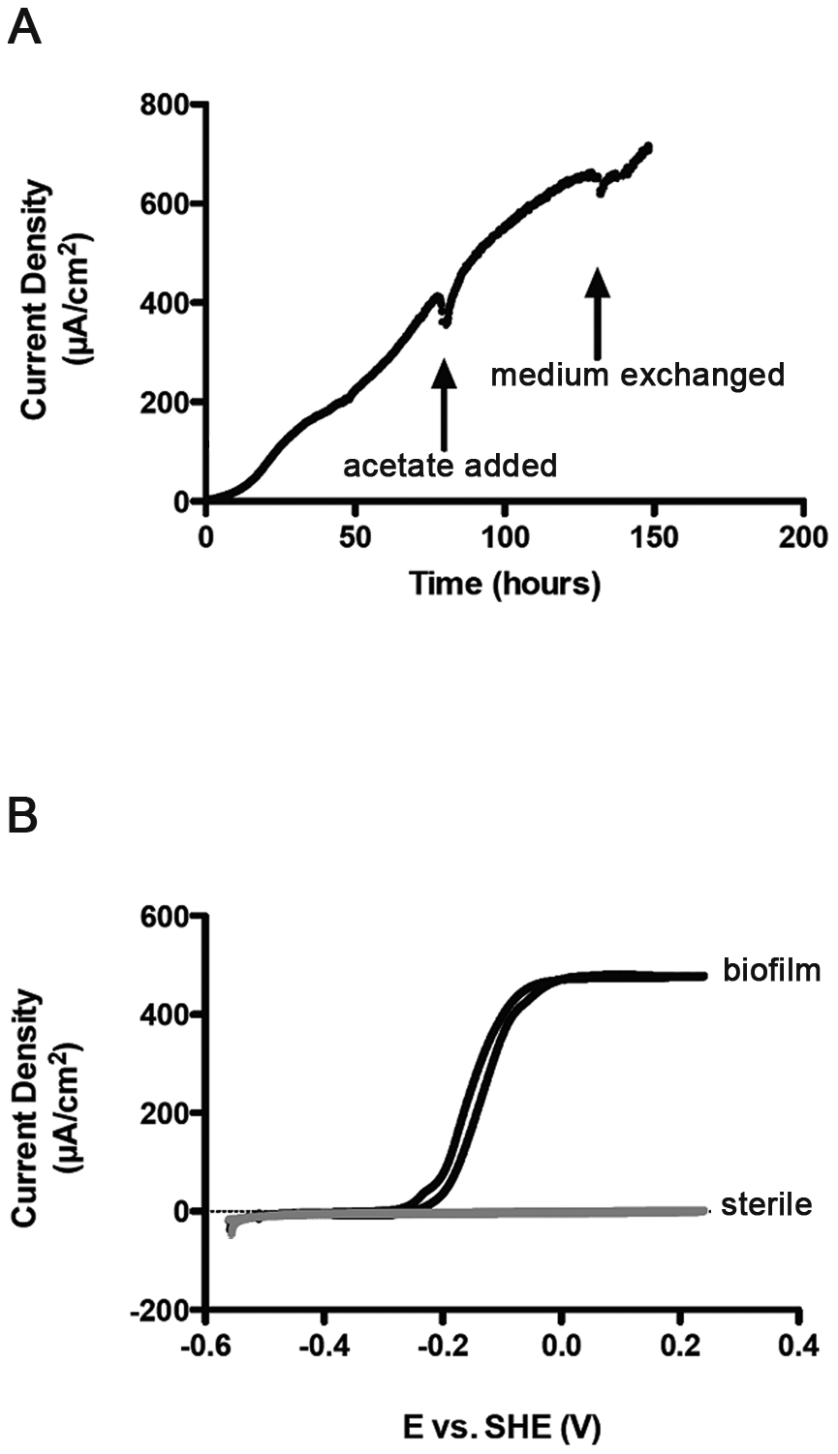
Growth of biofilms for analysis. (A) Representative trace showing current production by a *G. sulfurreducens* biofilm using 30 mM acetate as the electron donor on a polished graphite electrode poised at +0.24 V vs. Standard Hydrogen Electrode. Additional acetate (30 mM) was added where indicated, and the medium was replaced to remove planktonic cells. (B) Cyclic voltammetry (1 mV/s) of the electrode shown in A, producing the characteristic sigmoidal current response of a *G. sulfurreducens* biofilm-colonized electrode. All biofilms were grown simultaneously from the same inoculum under identical conditions, were harvested and fixed in resin at the same time.

*G. sulfurreducens* cells attached to poised electrodes (n = 8) with no lag, increased to a current density of >700 µA/cm^2^, and were all harvested at the same stage of growth (Fig. 1A). These growth rates and current densities were typical of *Geobacter* biofilms grown on polished graphite electrodes [8], [11], [32]. No biofilms demonstrated loss in current production when spent medium was removed and replaced with fresh medium. Cyclic voltammetry analysis yielded a sigmoidal catalytic wave with a characteristic midpoint potential (ca. −0.15 V) seen in growing *Geobacter* biofilms (Fig. 1B). Confocal microscopy of electrodes on which *G. sulfurreducens* biofilms were grown under similar conditions revealed biofilms of intact cells, based on Live/Dead staining, extending ∼20 µm from the electrode surface [8], [33], [34]. This thickness matched the resin-embedded slices imaged via TEM, indicating little shrinkage, collapse, or loss of the biofilm occurred during fixation.

**Figure 2:**
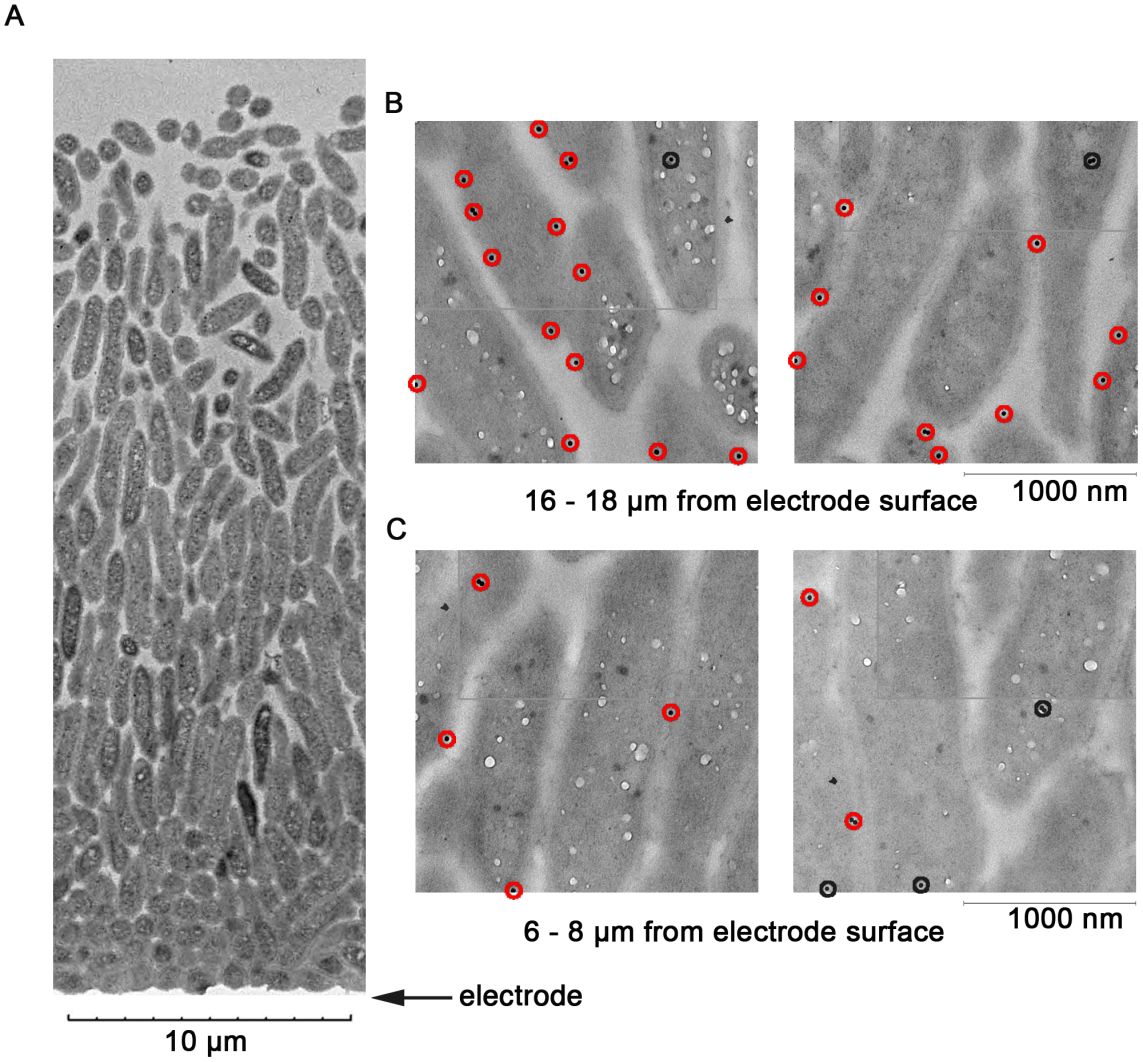
Representative biofilm and labeling with anti-OmcB antibody. (A) Low-resolution Transmission Electron Microscopy image showing an entire unlabeled *G. sulfurreducens* biofilm, illustrating increase in cell density near the electrode surface. (B) Examples from digitally reconstructed high-resolution images representing cells 16–18 µm from the electrode labeled with *anti*-OmcB antibodies. Red circles indicate proteins within 15 nm of the membrane, black circles indicate intracellular localization. (C) Examples of cells located 6–8 µm from the electrode, taken from the same biofilm reconstructions, with lower abundance of OmcB.

Fig. 2A shows a representative TEM micrograph of a *G. sulfurreducens* biofilm grown on the anodic electrode, spanning over 20 µm (the electrode is indicated by the arrow). The cells growing closer to the electrode were longitudinally oriented, and densely packed, causing the cell density near the electrode to be over 30% higher than in regions more distant from the electrode.

### Analysis of OmcB abundance in biofilms using immunogold labeling

After development of anti-OmcB antibodies and labeling conditions (see Methods), 70-nm thick biofilm slices were immunogold labeled and imaged via TEM. Two trends were immediately apparent in all images and were later substantiated by quantitative analysis. First, the majority of OmcB labeling was associated with the cell membrane, indicating that proteins embedded in membranes were exposed by microtome slicing. Second, cells farther from the electrode (10 µm to 20 µm) had a higher density of membrane-associated OmcB. Representative images are shown in Fig. 2B and 2C. Particles counted as membrane-associated (within 15 nm of a visible membrane) are indicated in red, and intracellular particles are highlighted in black. The distance of 15 nm for membrane association was based on the length of the rabbit IgG antibody (8.6 nm), plus the length of secondary antibodies [35].

To better quantify the abundance and localization of OmcB, a series of 12–15 high-resolution TEM images at 10,000× magnification were collected, and digitally re-assembled to produce a continuous picture of an entire biofilm. Each biofilm could then be divided into 2 µm sections, beginning from the electrode surface. Within each section, gold particles were separated into two categories; those inside the cell, and those at the membrane. A raw image showing the process of digital re-assembly using 13 high-resolution images is provided in Supplementary Information (Fig. S1). This sequence of slicing, high-resolution imaging, digital reconstruction, and abundance measurement was performed independently at least 3 times for OmcB quantification analyses.

**Figure 3:**
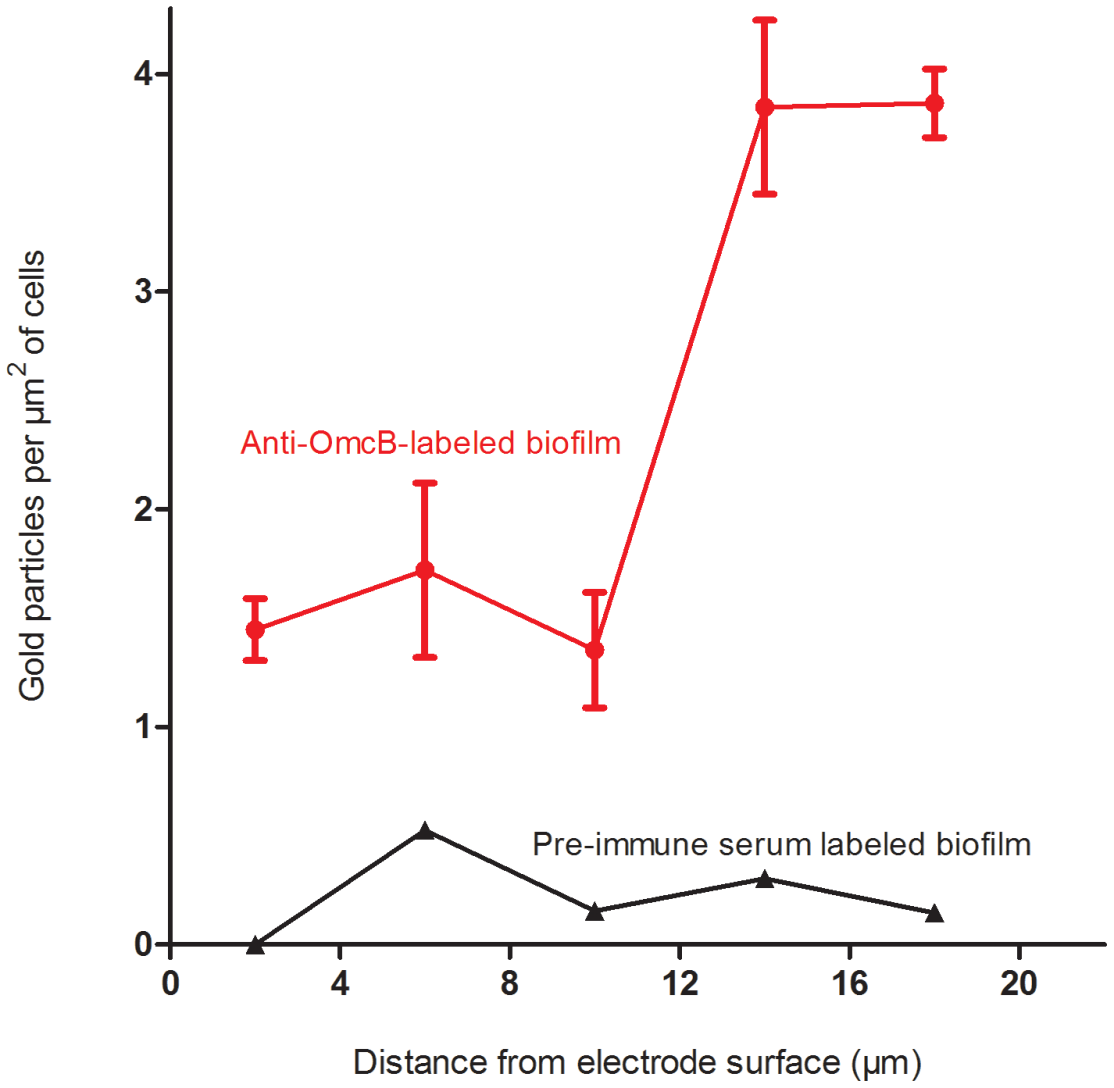
Abundance of OmcB on *G. sulfurreducens* cells at different distances from the electrode. Means shown are the result of six different images compiled from three different biofilms, +/− SEM. Data from labeling after incubation with *anti*-OmcB antibodies (red circles) vs. labeling with pre-immune serum as a control (black triangles).

Because cell density in the biofilm decreased away from the electrode, the number of gold particles had to be normalized for cell area present. Thus, the total abundance of OmcB (as total number of gold particles per µm^2^ of cells) could be expressed as a function of distance from the electrode and is shown in Fig. 3.

While some OmcB was always detected inside cells, presumably from apoproteins awaiting secretion, this baseline of intracellular OmcB labeling remained low throughout the biofilm (ca. 0.5 particles per µm^2^), with over 70% of the detected OmcB protein localized to the membrane in outer biofilm slices. However, the overall abundance of outer membrane-localized OmcB increased significantly with distance from the electrode. A range of 0.17 to 1.00 particles per µm^2^ was observed on cell membranes in the inner 10 µm of the biofilm, while a range of 2.15 to 3.42 particles per µm^2^ was observed on cell membranes in the outer 12–20 µm of the biofilm (n = 3).

Statistical analysis (ANOVA with two factor replication) of OmcB abundance in slices close to the electrode vs. outer leaflets, as well as in the membrane *vs.* inside the cells, was performed to verify these differences. For all biofilms analyzed, the increase in OmcB labeling of cells 12–20 µm away from the electrode was significant at p-values<0.001. The increased abundance of membrane vs. cytoplasmic labeling were significant at p<0.03 for all slices analyzed.

When the same labeling experiment was performed on slices using pre-immune serum, few particles were detected, and no trends in localization or abundance were observed. As this pre-immune serum was obtained from the same animals before exposure to the OmcB protein, and should not contain antibodies to OmcB, this represented a control for non-specific labeling of *Geobacter* cells.

### Analysis of acetate kinase immunogold labeling

**Figure 4:**
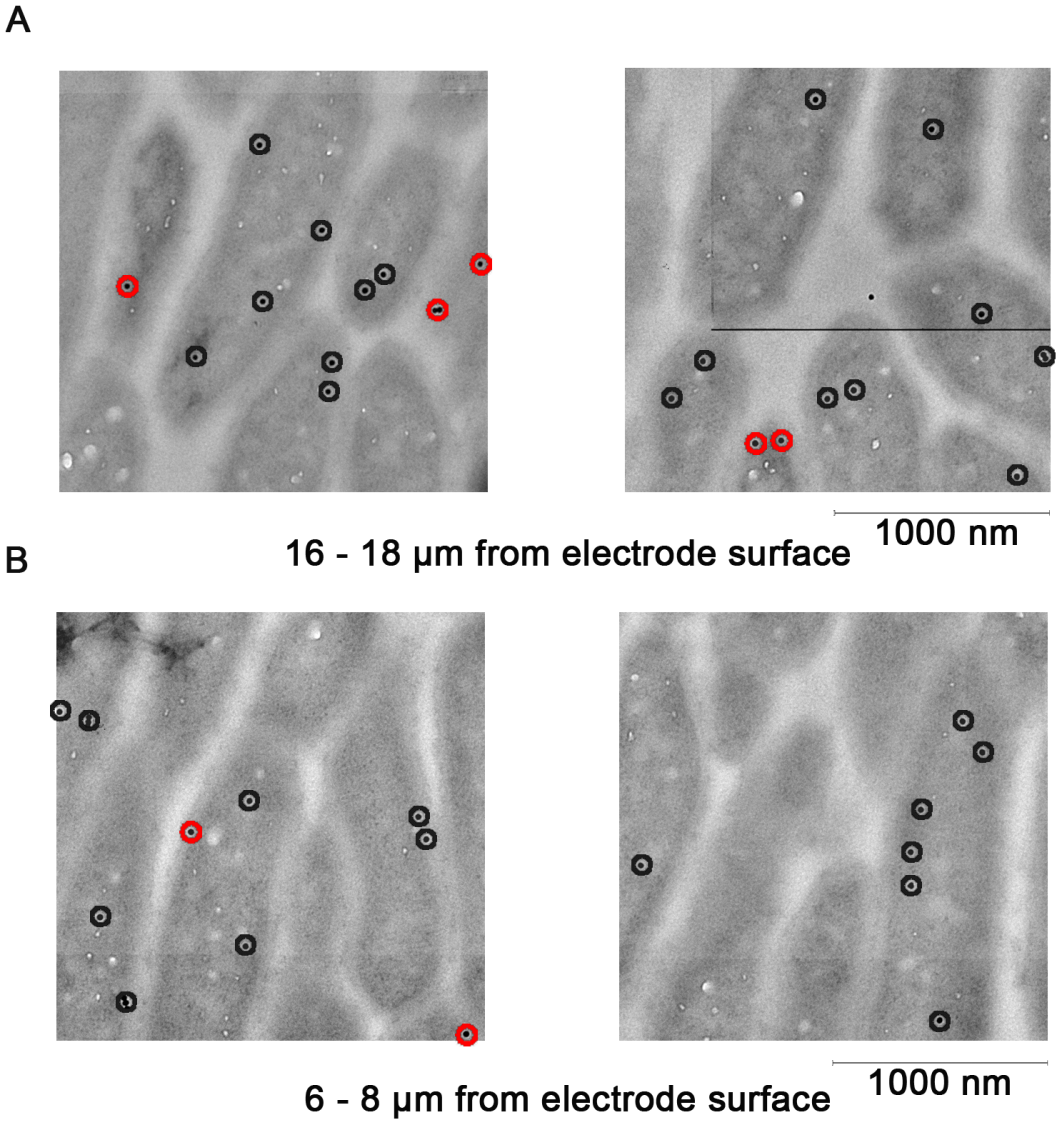
Anti-Acetate kinase labeling of *G. sulfurreducens* biofims. (A) Examples from digitally reconstructed high-resolution images representing cells 16–18 µm from the electrode labeled with *anti*-acetate kinase antibodies. Red circles indicate proteins within 15 nm of the membrane, black circles indicate intracellular localization. (B) Examples of cells located 6–8 µm from the electrode, taken from the same biofilm reconstructions.

**Figure 5:**
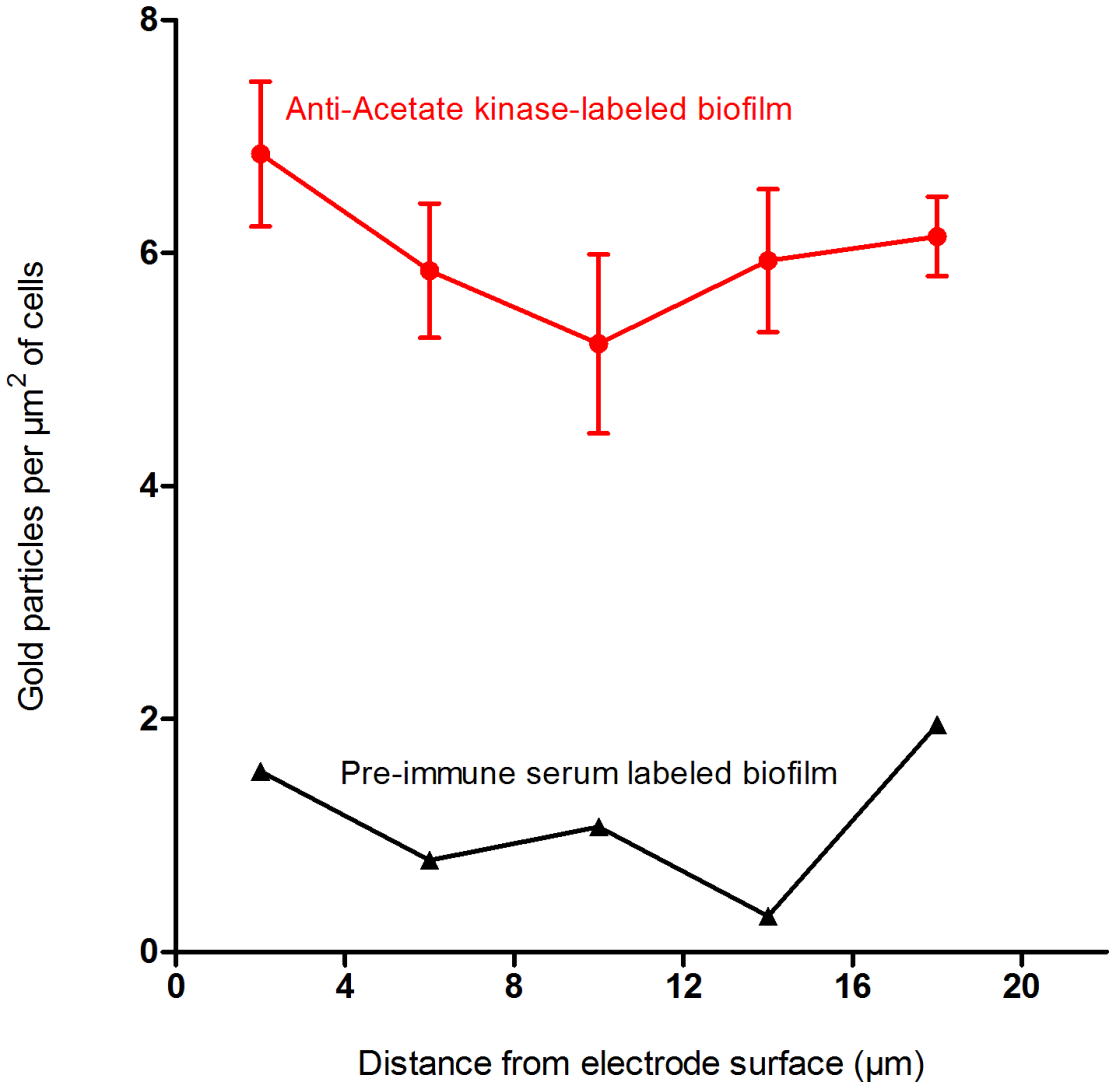
Abundance of acetate kinase isozymes at different distances from the electrode. Means shown are the result of six different images, +/− SEM. Data from labeling after incubation with *anti*-acetate kinase antibodies (red circles) vs. labeling with pre-immune serum as a control (black triangles).

Acetate kinase abundance was also analyzed by immunogold labeling, as a control for the ability to determine protein location, and as a marker for actively metabolizing cells. As with OmcB, multiple TEM images were assembled into contiguous sections from the electrode surface to the edge of the biofilm. Labeling inside cells was observed throughout the biofilm (Fig. 4A and 4B), and distance from the electrode had no effect on particle density. Cells 0–10 µm away from the electrode contained a range of 2.33 to 2.92 particles per µm^2^ of cells, while sections farther from the electrode contained 1.68 to 2.68 particles per µm^2^ of cells. In all regions of the biofilm, 78% of particles were detected on the interior of the cells, with very rare instances of labeling between cells which could indicate cell lysis or release of acetate kinase. Nonspecific labeling was also low for the acetate kinase pre-immune serum labeled biofilm (Fig. 5).

## Discussion

When *Geobacter* species oxidize acetate, they are absolutely dependent upon electron transfer to a terminal electron acceptor for energy generation. Compared to the use of metal oxide particles or soluble compounds, the electrode biofilm environment creates unique physiological challenges [23], [24], [36], [37]. In addition to the need to maintain a conductive network able to carry an ever-increasing burden when growing in cell layers more distance from the electrode, transfer of negatively charged electrons into the electrode creates a need for positive charge to diffuse outward to maintain both charge and pH balance [12], [17], [19], [23]. Much recent research has been focused on how, or if, *Geobacter* responds to these challenges.

While many multiheme *c*-type cytochromes may play roles in metal reduction [28], [38]–[43], one cytochrome that is conserved and consistently identified in studies with *G. sulfurreducens* is OmcB. OmcB is a putative lipoprotein dodecaheme *c*-type cytochrome which fractionates in the outer membrane fraction [44], [45], and has been shown via immunolocalization to be exposed on the outer surface [46]. Many mutant phenotypes in Fe(III) reduction can be traced to defects in *omcB* expression or translation [40], [47], [48]. For example, deletion of the gene encoding the diheme peroxidase MacA decreases *omcB* transcripts to undetectable levels and negatively impacts Fe(III) reduction, while expression of *omcB* from a constitutive promoter in a Δ*macA* mutant restores Fe(III) reduction [40], [47], [49]–[51]. As one of the most conserved cytochromes among the *Geobacteraceae*, and as the cytochrome most often linked to electron transfer beyond the cell membrane, OmcB was chosen as a target for this proof-of-concept study.

Changes in cytochrome expression by planktonic cells during Fe(III)- or electrode reduction have been reported using DNA microarray [52], [53] and proteomic [31], [42] approaches. However, as cells in a biofilm are not all exposed to identical conditions, such global analyses may represent averages that hide local microenvironments. We hypothesized that by assembling high-resolution images from biofilms growing on surfaces to minimize variability in cell age and distance from the electrode, while accounting for differences in cell density, quantitative data related to both cellular location and overall protein abundance per cell could be obtained.

In these experiments, the total abundance of OmcB was found to increase with distance from the electrode, a finding that could be due to two factors; differences in protein abundance per cell, or variability in antibody labeling. With regards to antibody labeling, thin resin-embedded cell sections are commonly used to quantify integral membrane proteins in different bacterial genera, even when cells are closely associated [53]–[57]. While methods that use flash-freezing can preserve delicate aspects of cellular ultrastructure, the antigenicity of target proteins is better preserved in resin-embedded samples, likely because freeze-substitution methods require use the chemical fixative osmium tetroxide, which affects protein antigenicity [55].

To minimize labeling artifacts in this study, slicing was used to give antigens equal probability of being exposed, compared to *in situ* labeling prior to slicing where cell packing or association limits access. Raw *G. sulfurreducens* images (provided in Fig. S1) demonstrated labeling at both cell-cell junctions and exposed membranes, providing supporting evidence that some antigens at the interface were accessible to antibodies, regardless of local conditions. While chosen for its reported lack of bias [58], the resin embedding-slicing method suffers from the fact that it must only label a very small percentage of proteins present in membranes, as each slice likely contains hundreds of OmcB targets, only a few of which are exposed at the interface. To compensate for this low efficiency, all embedding, slicing, and labeling steps were performed together, so that changes in the amount of detected protein could best reflect overall abundance.

If OmcB is more abundant in upper sections of the electrode-grown biofilm, then what could cells be responding to? Expression of *omcB* is affected by the stress-related sigma factor RpoS [59]–[61] and the stringent response [62], [63]. More relevant to growth in these biofilms, which were under constant electron donor levels and nutrient conditions, is the fact that expression of *omcB* increases when *G. sulfurreducens* is limited for Fe(III) as an electron acceptor [30], [64]. One hypothesis is that, as layers farther away from the electrode become more reduced, less electron acceptor is available in these regions, and OmcB increases in response. More generally, if the outer regions of the biofilm offer fewer opportunities for electron transfer, extra electron transfer machinery may be needed to achieve similar rates of electron disposal.

The concept that the outer portion of the *Geobacter* electrode biofilm represents a zone of electron acceptor limitation is not new. Spectral observations show nearly 50% of *c*-type cytochromes remain reduced when cells are growing in films similar to those used in this study [19], an observation also supported by confocal Raman spectroscopy [21]. Microelectrodes probing thicker (∼150 µm) *G. sulfurreducens* biofilms growing on electrodes showed that outer regions of the biofilm were at low redox potential, and oxidized zones were only detected in the inner portion of the film [17]. Source-drain measurements using *G. sulfurreducens* films grown across small gaps also showed that redox potential can be significantly lower only 10 µm away from an oxidizing electrode [12]. As OmcB appears to increase along the same spatial scales, this suggests that *Geobacter* can sense and respond to redox potential, in a manner similar to their known ability to sense Fe(III) availability.

In contrast to OmcB, acetate kinase labeling was consistent across the biofilm, providing no evidence for changes in acetate concentrations. As these cells were cultivated with high levels of acetate (30 mM), and acetate is never depleted below saturating concentrations in these thin biofilms, the finding of consistent acetate kinase levels was not surprising. Redox stains and viability stains [25], [27], have also provided evidence of active metabolism throughout biofilms of this thickness.

Overall, these results support use of immunogold labeling and biofilm reconstruction to simultaneously study both protein localization and abundance with high resolution. In this initial experiment, a difference in OmcB abundance was detected, and the protein was associated with cell surfaces even in biofilms where extensive extracellular connections are known to form. Experiments are needed that apply this approach to different growth conditions, such as changes in electrode redox potential. These experiments could also target proteins hypothesized to be secreted into the space between *Geobacter* cells, which offer opportunities for calibration against other *in situ* labeling methods, and provide a quantitative understanding of how the conductive matrix changes to support extracellular respiration in different regions of the biofilm.

## Experimental Procedures

### Bacterial growth conditions

*Geobacter sulfurreducens* PCA was routinely grown with Fe(III) oxide (100 mM) as the electron acceptor and 20 mM acetate as the electron donor, and transferred 5 times into mineral media with 20 mM acetate and 40 mM fumarate when cells were needed for electrode growth [8]. All incubations were performed at 30°C under a 20% CO_2_/80% N_2_ atmosphere. The bioreactor consisted of a jacketed glass electrochemical cell (Pine Instruments, Raleigh, NC) fitted with custom Teflon stoppers, sample ports and gas inlets. Working electrodes consisted of 2 cm^2^ AXF-5Q graphitic carbon electrodes (Poco Graphite Company, Decatur, TX) that were polished with a 0.05 µm alumina slurry (BASi, West Lafayette, IN), sonicated in deionized water, and cleaned with acetone, 1 M NaOH, 1 M HCl, and rinsed with deionized water between each step. Eight graphite electrodes were suspended on 0.25 mm platinum wires (Sigma-Aldrich, St. Louis, MO) that were soldered to copper wire heat-sealed inside 3 mm glass capillary tubing (Kimble, Vineland, NJ). Counter electrodes of Pt were also constructed in this manner. A Vycor frit-tipped Luggin tube with a 0.1 M Na_2_SO_4_ 1% agar salt bridge housed a calomel reference electrode (Cole-Parmer, Vernon Hills, IL). Electrodes were placed in stirred reactors, and connected to channels of a VMP potentiostat (Bio-Logic, Knoxville, TN).

Cells were cultivated in medium with excess electron donor until the optical density at 600 nm reached 0.6, and 60 mL was transferred into the autoclaved reactor containing 60 mL of mineral media with 30 mM acetate. The electrodes were poised at 0.24 V *versus* standard hydrogen electrode (SHE), and 30 mM additional acetate was added at 80 hours. Fresh medium with 30 mM acetate was added as current plateaued (131 hours) to remove planktonic and loosely attached cells. Cyclic voltammetry was performed at 1 mV/s from −0.56 to 0.24 V vs. SHE on sterile reactors, and at various time points during growth. The biofilm-bearing electrodes were harvested at 150 hours, gently rinsed in sterile media and fixed in a solution of 3% paraformaldehyde and 0.05% glutaraldehyde buffered with 0.05 M sodium phosphate at pH = 6.8.

### Preparation of biofilms for TEM

After fixation, the biofilms were embedded in L.R. White Resin and the resin was allowed to polymerize. The biofilms were sectioned at the Penn State Microscopy and Cytometry Facility (University Park, PA) into vertical sections, so that each slice spanned the entire biofilm. The 70 nm thick sections were floated onto copper grids (carbon coated) and permitted to dry before immunolabeling.

### Cloning and heterologous expression of OmcB

The full length *omcB* gene (2235 bp) was amplified via PCR from *G. sulfurreducens* PCA genomic DNA. The primers used for amplification were OmcB forward primer: (GCTAGC ATGAGTAGAAAAGTAACAAAGTAT) and OmcB reverse primer: (CTCGAG CGGACGGGTCGT), the NheI and XhoI restriction enzymes sites are underlined, respectively. The gene was sub-cloned into pGEM T-easy vector (Promega) and DH5α competent cells. Colonies were selected on ampicillin, IPTG and X-Gal plates (AIX plates) incubated at 37°C and then inoculated into Luria-Bertani (LB) medium with 100 µg/mL ampicillin to be screened for the *omcB* gene. Plasmid DNA was isolated from each culture using Macherey-Nagel Nucleospin kit and digested with NheI and XhoI restriction enzymes. The *omcB* gene was gel extracted and ligated into the pET21a(+) expression plasmid (Novagen) to produce a His-tag fusion protein through heterologous expression in BL21 (DE3) pEC86 competent cells. The pEC86 plasmid [65] contains genes required for heme maturation and also confers chloramphenicol resistance.

The transformed cells were selected on solid LB medium (incubated at 37°C) containing 200 µg/mL ampicillin and 10 µg/mL chloramphenicol. Colonies were selected and inoculated into LB medium containing 100 µg/mL ampicillin and 35 µg/mL chloramphenicol antibiotics and grown overnight at 37°C. For screening of OmcB expression, 1 mL of the cells were used as an inoculum in 11 mL LB medium with 100 µg/mL ampicillin and 35 µg/mL chloramphenicol antibiotics and grown at 37°C. Protein expression was induced with 1 mM IPTG when the optical density of the cells reached 0.6 at 600 nm. Induced cells were grown for 3 hours with shaking at 37°C. Expression was visualized on SDS-PAGE gels stained with Coomassie R-250 stain. Cells containing the *omcB*-pET21a plasmid were stored as glycerol stocks in a −74°C freezer.

The full length *omcC* gene (2307 bp) was also amplified and expressed as described for the *omcB* gene. The forward primer sequence was: GCTAGC ATGAGTAGAAAAGTAACAAAGTAT and the reverse primer sequence was: AAGCTT CGGACGGGTCGC. The NheI and HindIII restriction enzyme sites are underlined, respectively.

### Purification of recombinant OmcB

For over expression and purification of the recombinant OmcB protein, *E. coli* cells harboring the *omcB*-pET21a plasmid were inoculated into 500 mL of LB medium containing 100 µg/mL ampicillin and 35 µg/mL chloramphenicol and grown and induced as previously described. The cells were harvested by centrifugation at 7,311×g for 10 minutes at 4°C and stored at −74°C. Purification was via a procedure modified from that used by [66]. The cell pellet was resuspended in 0.4 volumes of 50 mM Tris-Cl, pH 8 with 2 mM EDTA and 10 mM DTT. Next, 0.01 volumes of 10 mM PMSF in 90% isopropanol, 10 mg/mL lysozyme and 1% Triton X-100 were added to the cell suspension. The cell suspension was incubated at 30°C for 30 minutes and the cells sonicated five times for 1 minute each time, on ice. The cell suspension was then centrifuged at 7,311× g for 20 minutes at 5°C. The pellet was stored at −20°C. The pellet was resuspended in just enough 50 mM Tris-Cl, pH 8 buffer with 8 M urea, 2 mM ETDA and 1 mM DTT to get the pellet into solution. A homogenizer was used to fully resuspend the pellet in the buffer.

The pellet solution was applied to a Ni-NTA column (Qiagen) equilibrated with the binding buffer (100 mM NaH_2_PO_4_, 10 mM Tris-Cl and 8 M urea, pH 8). The flow through fraction was collected and the column washed with 3 volumes of the wash buffer (100 mM NaH_2_PO_4_, 10 mM Tris-Cl and 8 M urea, pH 6.3). The wash fraction was collected and the OmcB protein that was bound to the column was eluted with 10 mL of elution buffer (100 mM NaH_2_PO_4_, 10 mM Tris-Cl, 8 M urea and 0.5 M imidazole, pH 8). All three fractions were then resolved on a 12% SDS-PAGE. Coomassie staining of the gel revealed that the eluted fraction consisted mostly of the OmcB protein. The eluted fraction was then diluted with distilled H_2_O (to dilute the 8 M urea) and concentrated in an Amicon stirred cell with a 10 kDa cutoff (Millipore) at 4°C to a volume of 1 mL. The concentrated, purified protein was dialyzed at 4°C in 5 mM NaH_2_PO_4_ buffer, pH 7. One exchange of buffer was performed and the pure protein was stored at −20°C. Removal of the urea resulted in some precipitation of OmcB. The protein concentration was determined via the Lowry assay [67] with BSA as the standard.

### Peptides of acetate kinase for antibody preparation

Two peptides for labeling both acetate kinase isozymes were selected from the protein sequences using the hydropathicity plots program on www.vivo.colostate.edu (Colorado State University). The peptides selected, RRDVIEHASNGDHRC and CIEGLEGIGIKLDRERNKGAM, are hydrophilic and putative antigenic regions of the protein, suitable for antibody production. The sequences were then compared to other protein sequences on the non-redundant protein database through NCBI Protein BLAST program. The peptides only matched acetate kinase in *G. sulfurreducens* PCA with the first peptide matching AckA-1 (GSU2707) and the second peptide matching AckA-1 and AckA-2 (GSU3448). The seqeunce of the second peptide matching AckA-2 was GIKLDRERN and the lysine residue was substituted with an arginine residue in AckA-2. A cysteine residue was placed at the amine terminus of the second peptide so that it could be linked to a carrier protein for antibody production. Both peptides were synthesized by the Penn State Hershey Macromolecular Core Facility.

### Antibody production

Purified OmcB protein and acetate kinase peptides were sent to Covance Custom Immunology Services Inc. (Princeton, NJ) as antigens for antibody preparation. Antibodies were made in New Zealand White rabbits. Antibodies to OmcB were affinity purified as previously described [68]. The affinity purified antibody was kept at −20°C.

The acetate kinase peptides were covalently linked to a Sulfolink Coupling Resin (Pierce Biotechnology, Rockford, IL) at the cysteine residue and used to affinity purify the acetate kinase antibodies. The peptides were linked to the Sulfolink resin based on the manufacturer's instructions. The acetate kinase antibodies were purified based on the procedure described in [69]. The OmcB and acetate kinase pre-immune sera were purified using Protein A agarose resin (Sigma Aldrich) according to the manufacturer's procedure. All antibody concentrations were determined using the Bradford assay [70].

### Detection of OmcB and Acetate kinase

Proteins from *G. sulfurreducens* whole cells or from the total membrane (TM) were separated on a SDS-PAGE and transferred to nitrocellulose for Western blotting with a 1∶100 dilution of anti-OmcB affinity-purified antibody or 1∶1000 dilution of anti-acetate kinase affinity-purified antibody. The polyclonal OmcB antibody produced only one cross-reactive band when 10 µg of *G. sulfurreducens* total membrane protein was analyzed. *E. coli* expressing either OmcB or OmcC, a slightly larger homolog of OmcB which has 73% amino acid identity, was also analyzed via Western blotting. While OmcB was easily detected with the OmcB antibody, ten times more protein had to be loaded for Western blots to reveal a faint band for OmcC. Densitometry of the Western blots with calibrated levels of protein indicated the OmcB antibody had over 30-fold higher affinity for OmcB compared to OmcC.

As acetate kinase was intended to act as a control for cytoplasmic antibody labeling, synthetic peptides were used to generate an antibody able to recognize both acetate kinase isozymes expressed by *G. sulfurreducens*. Western blots of cells detected both forms, at 47 kDa and 44 kDa, with the major band being the isozyme of greater molecular weight. Previous proteomic data also reported significantly higher abundance of this isozyme under all growth conditions [31].

OmcB and acetate kinase pre-immune sera were also incubated with membrane proteins and whole cells, respectively, and did not show a band corresponding to OmcB or acetate kinase under any conditions. These pre-immune sera were used as controls for nonspecific labeling of biofilms.

### Immunolocalization of OmcB and acetate kinase in biofilm

Each carbon-coated copper grid containing a *Geobacter* biofilm slice (spanning the whole biofilm, top to bottom) was allowed to incubate sample-side down on a drop of TBS (10 mM Tris-Cl, pH 8, 150 mM NaCl), for 1 minute. Each grid was then placed on a drop of 0.3% glycine in TBS for 5 minutes and then on a drop of 1% BSA in TBS for 30 minutes. After this blocking step, each grid was incubated on a drop of 1∶50 dilution of OmcB antibody (0.6 µg total protein) in 1% BSA in TBS or on a drop of 1∶100 dilution of acetate kinase antibody (4 µg total protein). Separate grids incubated on purified pre-immune serum for OmcB and acetate kinase were used as controls. The grids that were used in the OmcB immunolabeling experiment were incubated in the antibody or pre-immune serum for 10 hours at 4°C, based on preliminary immunolabeling experiments with the OmcB antibody that showed long incubations at low temperature increased labeling without increasing non-specific binding by pre-immune serum controls. The grids for acetate kinase were incubated in the antibody or pre-immune serum at room temperature for 1 hour.

Each grid was then washed by placing it on a drop of TBS for 3 minutes (3 times) and then on a drop of 1% BSA in TBS for 3 minutes (5 times) to block the biofilm before labeling with the secondary antibody. Each grid was then allowed to incubate on a drop of the secondary antibody: goat anti-rabbit IgG secondary antibody conjugated to 20 nm gold particles (BB International), which was prepared as a 1∶100 dilution in 1% BSA and 0.1% cold fish gelatin in TBS. The secondary antibody labeling was carried out for 1 hour at room temperature. Each grid was washed by placing it on a drop of TBS as described above and then the biofilms were fixed by incubating them on a drop of 1% glutaraldehyde in TBS for 5 minutes. After this step, each grid was washed by placing it on a drop of water for 3 minutes (7 times) and then negatively stained on a drop of 2% uranyl acetate in water for 5 minutes in the dark. Finally, each grid was washed on a drop of water for 1 minute (5 times) and allowed to dry completely (at least 12 hours) before TEM imaging.

### TEM imaging and analysis of biofilms

Immunolabeled biofilms were visualized using a JEOL 1200 EX II transmission electron microscope (Pennsylvania State University, University Park) at 80 kV and a magnification of 10,000. A series of TEM images were reconstructed into continuous biofilm pictures using Adobe Photoshop CS2. The Image J software (NIH) was then used to take subsamples of each biofilm, representing 2 µm increments from the electrode surface. For each antibody and analysis, three complete biofilms were reconstructed.

Image J was also used to measure cell area within each slice with the Measure feature. The cell area values obtained from Image J were converted to µm^2^ values by determining the length of the 1 µm scale bar in the TEM micrographs. The number of gold particles present inside cells, and at the membrane was counted using the Cell Counter plugin of Image J.

## Supporting Information

Figure S1 **Representative raw image.** Raw data from one complete set of 13 high-resolution images spanning an entire biofilm. Slices were labeled with *anti*-OmcB antibodies post-slicing. From this raw data, trends in both cell density, OmcB labeling, and protein localization can be seen before images were analyzed further. After this reconstruction, digital images were separated into fields encompassing distances from the electrode discussed in the text and figures, and relative cell volume and labeling measured.
(TIF)
