# Supplementary figures and images for: Abundance of the Multiheme c-Type Cytochrome OmcB Increases in Outer Biofilm Layers of Electrode-Grown Geobacter sulfurreducens
Source: PLoS One. 2014 Aug 4;9(8):e104336. doi: 10.1371/journal.pone.0104336 (PMC4121341; doi:10.1371/journal.pone.0104336)

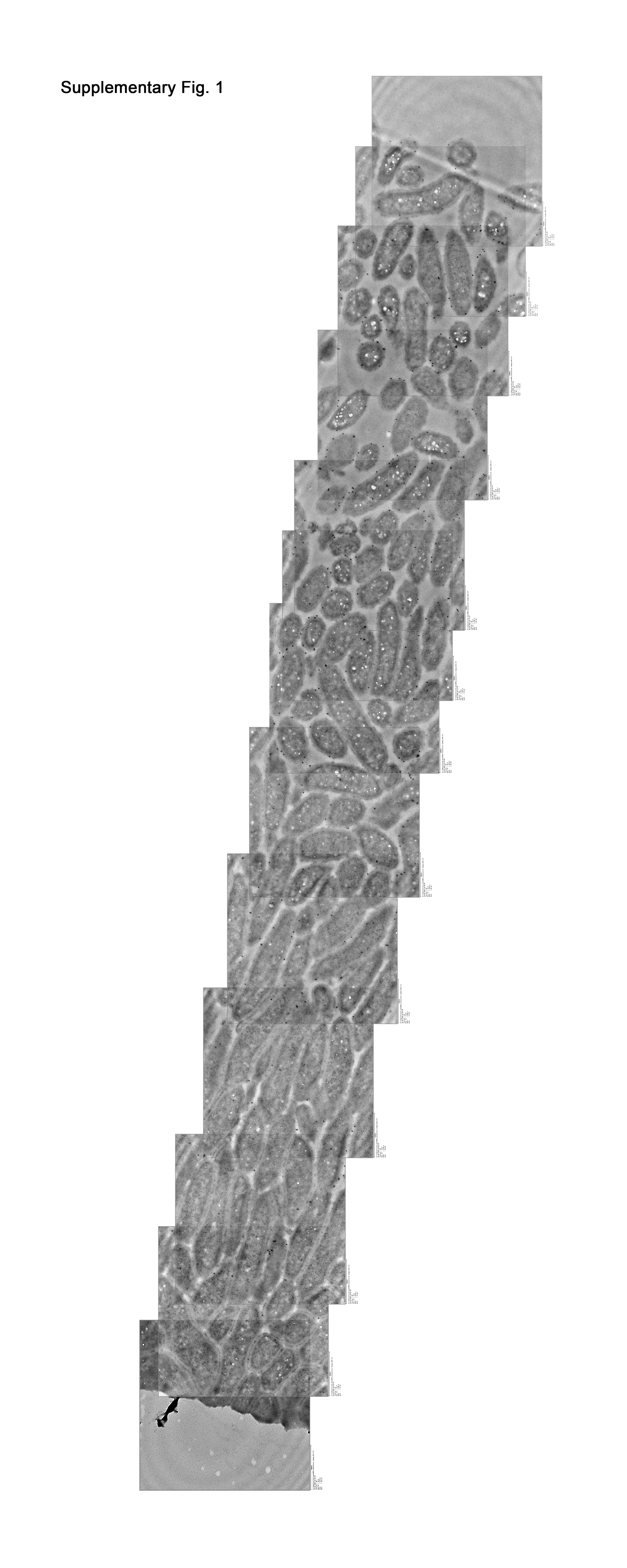

Supplement: Figure S1 — Representative raw image. Raw data from one complete set of 13 high-resolution images spanning an entire biofilm. Slices were labeled with anti-OmcB antibodies post-slicing. From this raw data, trends in both cell density, OmcB labeling, and protein localization can be seen before images were analyzed further. After this reconstruction, digital images were separated into fields encompassing distances from the electrode discussed in the text and figures, and relative cell volume and labeling measured. (TIF) [file pone.0104336.s001.tif]
